# Rapid, label-free pathogen identification system for multidrug-resistant bacterial wound infection detection on military members in the battlefield

**DOI:** 10.1371/journal.pone.0267945

**Published:** 2022-05-05

**Authors:** Ying Chen, Julie Chau, Jung Yoon, Jeanne Hladky

**Affiliations:** Optowares, Inc., Woburn, Massachusetts, United States of America; Universidade Católica Portuguesa Escola Superior de Biotecnologia: Universidade Catolica Portuguesa Escola Superior de Biotecnologia, PORTUGAL

## Abstract

US military service members experiencing combat-related wounds have higher risk of infection by multidrug-resistant bacteria. The gold standard culture-based antimicrobial susceptibility testing (AST) is not feasible in the battlefield environment. Thus, a rapid deployable system for bacteria identification and AST directly from wound sample is urgently needed. We report the potential of a Rapid, Label-free Pathogen Identification **(**RAPID) diagnostic system based on ATR-FTIR method to detect and distinguish multi-drug resistant strains for six different species in the *ESKAPEE* group. Our RAPID system combines sample processing on-broad to isolate and enrich bacteria cells from wound sample, ATR-FTIR measurement to detect antimicrobial-induced bacterial cell spectral changes, and machine learning model for automated, objective, and quantitative spectral analysis and unknown sample classification. Based on experimental results, our RAPID system is a promising technology for label-free, sensitive (10^4^ cfu/mL from mixture), species-specific (> 95% accuracy), rapid (< 10 min for identification, ~ 4 hours for AST) bacteria detection directly from wound samples.

## Introduction

### Challenge: Lack of field-capable assays to rapidly identify multidrug-resistant bacteria in combat-related wound infection

US military service members who are evacuated from the battlefield due to injuries had likely sustained traumatic insults such as explosions, gunshot wounds and vehicle accidents. They have higher chance of developing infection while waiting for transfer and after admission to military medical care facilities due to extensive skin and soft tissue injuries [1–3]. Studies had reported that wound infections occur in 17% - 25% of combat-related injuries in the US military treatment facilities. [4–6]. Among these infections, 14% - 22% are found to be caused by multidrug-resistant (MDR) bacteria [7–9]. For example, *Acinetobacter baumannii* has been identified as one of the most common pathogens in combat-related injuries associated with skin and soft tissue (35%). Within this 35%, up to 90% culture isolates were determined to be MDR [10]. Community-acquired methicillin-resistant *Staphylococcus aureus* (CA-MRSA) is another well-recognized MDR in traumatically injured service members (68% - 70%) in selected military hospital emergency rooms [11].

MDR infections in combat wounds could lead to higher amputation rate, more subsequent operations, greater risk for developing chronic infection, osteomyelitis and sepsis, and readmission or extended stay in the medical facility [10,12]. Furthermore, treatments of MDR infections on combat-related wounds requires high medical resource consumption and are reportedly less successful compared to civilian wounds [13,14]. Therefore, as preventive measures, the patient is often given broad-spectrum antimicrobial treatment upon arrival to the medical facilities, without knowing the pathogen identity or the antimicrobial susceptibility profiles [15,16].

The gold-standard culture-based antimicrobial susceptibility testing (AST), where several rounds of cultivation are required (such as an enrichment round to increase number of bacteria, and a plate round to obtain pure culture) before testing with various antimicrobials [17], is not feasible in the battlefield. Culture-based AST needs to be conducted under aseptic condition in the laboratories by specialists and uses reagents that requires cold storages, which may not be workable due to logistic and manpower constrains in an austere battlefield environment. Furthermore, the turnaround time for the culture-based AST is slow (~ days) and thus not feasible for routine sampling to monitor patient progress.

Molecular method such polymerase chain reaction (PCR) is a promising alternative to the culture-based AST. PCR screens for the presence of known genes that confer antimicrobial resistance [18,19]. Commercial systems like Vitek® and Microscan® can perform turbidity measurements for multiwell liquid cultures in automated fashion, with typical turnaround times as short as 4 hours for ID and 6–8 hours for susceptibility testing [20,21]. However, these systems typically require positive culture bottles that have to be cultivated from patient samples prior to performing susceptibility testing, prolonging the total sample-to-answer time. Moreover, PCR-based method is unable to detect all antimicrobial resistance because many phenotypically resistant strains exhibit resistances due to a combination of mechanisms such as enhanced beta-lactamase production, membrane impermeability due to loss of porins and expression of efflux pumps on their cell membrane [22–25].

Global emergence of MDR pathogens is threatening the availability and effectiveness of antimicrobials [26–28]. As the trend of drug resistance emerging at faster pace than new FDA-approved antimicrobials is unlikely to be reversed in the foreseeable future [29,30], it is paramount to preserve the effectiveness of currently available antimicrobials. Early diagnosis of the pathogen is the key for allowing conservative and effective use of antimicrobials [31–33]. Unfortunately, there is currently no FDA-cleared, label-free, deployable, soldier-friendly, stable (no cold-storage requirement) and rapid (<30 minutes) for bacteria identification and AST in the field environment. Therefore, a rapid diagnostic system that can be used in modern battlefield environment to triage injured military service members and guide clinical decisions is urgently needed.

### Rapid Label-free Pathogen Identification (RAPID) for MDR bacteria detection

We are reporting the potential of our RAPID system based on ATR-FTIR (Fourier-transformed Infrared Spectroscopy with Attenuated Total Reflection modality) technique to fulfil the need for sensitive and specific detection of MDR bacteria strains in the field environment. Our RAPID system leverages off the commercial availability of portable, lightweight (~3 lb), battery powered FTIR spectrometer for use in food analysis, biodiesel monitoring, gemstone analysis and hazardous chemical identification [34–37] in the laboratory or field environment, and the recent advancements in multi-well ATR system for high-throughput studies [38]. When combining with our patent-pending, filtration-based sample processing method to enrich intact bacterial cells from wound samples and our machine-learning based analysis software package, our system can detect the presence of bacterial cells in less than 10 minutes and distinguish MDR strains in about 4 hours in the field, at as low as 10^4^ cfu/mL concentration from wound sample.

FTIR has a long history as a versatile analytical technique for label-free micro-organism detection and identification [39,40]. FTIR spectrum of a given molecule is highly specific due to the dense chemical information contained in a single spectrum. FTIR is a sensitive method for analyzing real-world bacterial samples, with a detection limit between 10^3^–10^5^ cfu/mL [41,42], which is several orders of magnitude lower than average bacteria concentration found in the pus drainage from infected wound (10^7^ to 10^8^ cfu/mL) [43,44]. However, the range of concentration and impurities FTIR will tolerate are narrow [45]. The presence of bulk water is extremely problematic for FTIR transmission measurements. Thus, samples need to be prepared into a thin, dried pellet, making FTIR a less user-friendly process.

Attenuated total reflection (ATR) was invented as a sampling technique to bring FTIR to a wider variety of samples with various contaminants and optical densities. In ATR the infrared light propagates within an ATR element via total internal reflection. Since the IR radiation is not transmitted through the bulk of the sample, the sample does not need to be prepared as a thin pellet. The limited depth of interaction (1–2 μm) of the IR radiation beyond the ATR crystal surface makes possible for collecting high-quality spectra in the presence of bulk water, while remain sufficiently deep to cover a monolayer of bacteria on the crystal surface. Moreover, most modern ATR instruments are capable for acquiring time-dependent spectra by repeatedly interrogating the ATR system and with software design to track intensity changes over time, giving ATR the ability for continuous monitoring for chemical/biological activities of the bacteria.

Many examples of how the ATR sampling technique enables micro-organisms detection by FTIR in complex aqueous environment had been reported in literature. For example, Wijesinghe *et al* constructed a low-cost, benchtop ATR-FTIR to detect antimicrobial resistance in *E*. *coli* [46]. ATR had been used to discriminate bacteria using whole organism fingerprinting [47], to monitor growth and cyclic changes in bacterial biofilm population [48,49], and to study penetration of antimicrobial agents into the bacterial biofilm [50,51].

In this report we describe the capability of our ATR-FTIR based system to detect and distinguish six different MDR and six antimicrobial-susceptible bacteria strains from spiked samples under laboratory environment. We demonstrated the strain-specificity, sensitivity and selectivity of our ATR system in recognizing unique bacterial spectral features in pure solution and in complex mixture through quantitative spectral analysis, and the use of machine learning models to automate the analysis process.

## Materials and methods

### MDR and antimicrobial-susceptible bacterial strain procurement

**Fig 1:**
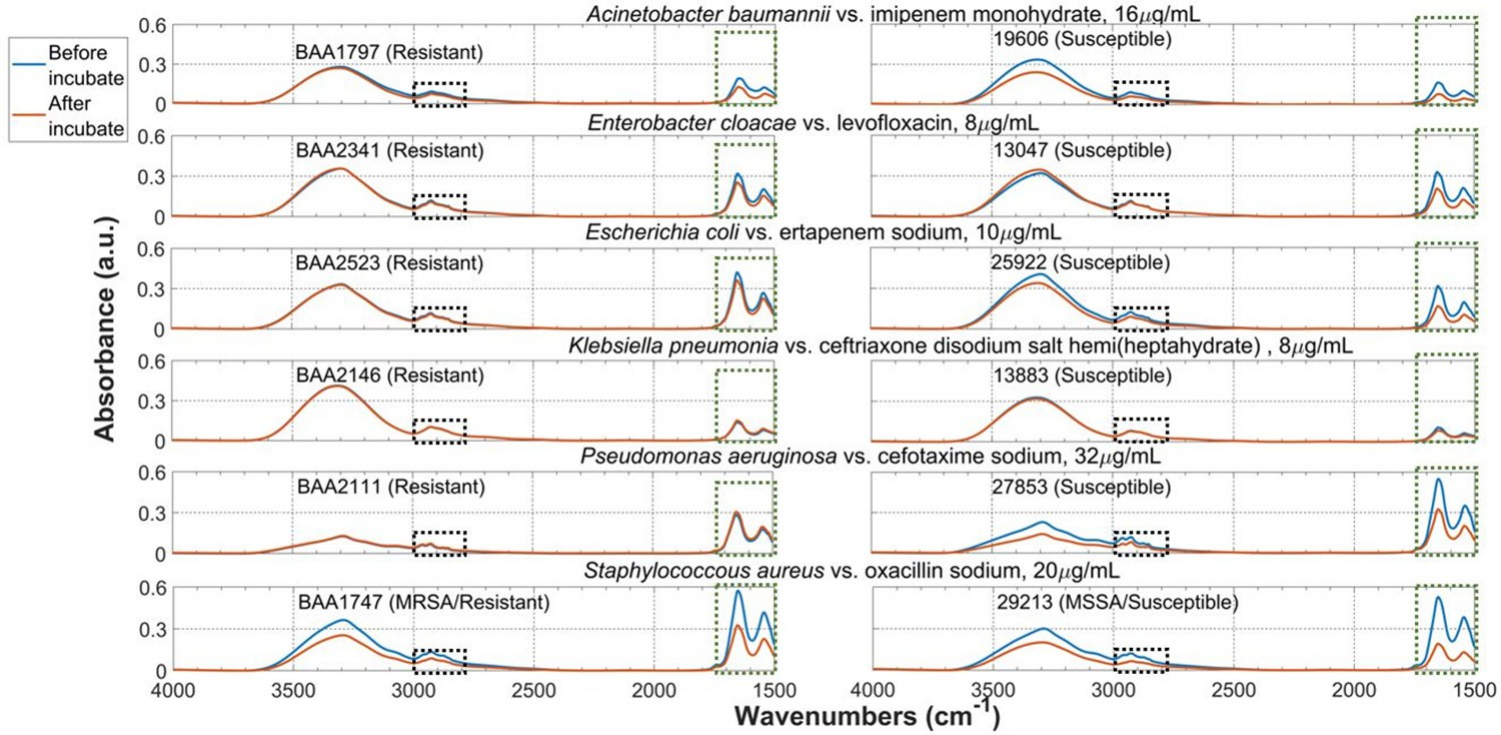
Specificity of the ATR system. Compare averaged ATR spectra of the bacteria cell before and after incubation with antimicrobial solution at 35°C for 4 hours. Consistent spectral changes are observed in two highlighted spectra regions.

Six MDR and six antimicrobial-susceptible strains were selected for this study. These 12 strains represented six different species from the *ESKAPEE* group–the acronym for a group of seven MDR species on World Health Organization Critical Priority I and II Pathogens lists: *Staphylococcus aureus*, *Klebsiella pneumoniae*, *Acinetobacter baumannii*, *Pseudomonas aeruginosa*, *Enterobacter cloacae* and *Escherichia coli*. The strain ID and susceptibility profile to six antimicrobials (one antimicrobial per species) are summarized in the table in Fig 1. All 12 strains were obtained from ATCC. These strains were cultured, harvested and stored according to ATCC guideline in our BSL-2 facility. We used the antimicrobial susceptibility profile and MIC_50_ values reported by ATCC, from the CLSI-M100 tables and literature for the strain without further verification. Bacterial samples emanated from the virgin ATCC samples were used in all the experiments (i.e. subcultured sample were not used).

Sterile, disposable, untreated cell culture flasks with 0.22 μm filter cap (Culture Area: 75 cm^2^, VWR®) with 60–75 mL broth were used to rehydrate the sample. Each stain was emanated for 18–24 hours with constant agitation in tryptic soy broth at 37°C, except for *Klebsiella pneumoniae* 13883 which required nutrient broth. After incubation, the bacterial cells were harvest by transferring the flask content to multiple sterile, 50 mL conical tubes and centrifuge at 8000 rpm for 10 minutes to obtain cell pellet. The cell pellet is aliquoted to multiple 1.5 mL cryogenic vials with 20% skim milk as cryoprotectant. The vials were stored in the vapor phase of a liquid nitrogen dewar.

Before centrifugation, 1 mL was sampled from the flask to determine the number of viable cells by quantitative cell culture. The procedure is following. The approximate cell concentration in the sample was estimated based on the cloudiness. Based on the estimation the 1 mL sample was serially diluted with tryptic soy broth or nutrient broth until reaching 10^−10^ to 10^−12^ (undiluted sample = 10^0^) level. Minimally 5 most diluted sample in the serial dilution were plated. The averaged number from these five plates was reported as the number of viable cells in the sample, typically in the 10^11^−10^13^ cfu/mL range.

### ATR-FTIR measurement setup

We used a Nicolet® iS10 FTIR spectrometer (ThermoFisher®) with a custom-built ATR accessory based on the horizontal ATR accessory (HATR, PIKE Technologies®) to obtain all ATR spectra in this report. The enclosure of the HATR accessory was removed to allow greater access to the ATR element. Both the HATR accessory and the FTIR spectrometer were under constant nitrogen purging to minimize absorption due to atmospheric water and CO_2_. Both HATR accessory and the ATR element are disinfected by 70% ethanol/water between each measurement.

A multi-bounce silicon 45° trapezoidal ATR element (80 x 10 x 2 mm L x W x H, PIKE Technologies®) was used in all ATR measurements. We selected Si because of its hardness and chemical resistance to 70% ethanol/water compared to other ATR element materials. An air background spectrum was collected before each ATR measurement. The bacterial ATR spectra were acquired and analyzed using OMNIC® Software (ThermoFisher®) with the following setting:

Spectral region recorded: 4000–1500 cm^-1^. Spectral Resolution: 4 cm^-1^. Atmospheric suppression: On. # of scans: 256.

### Bacterial sample preparation

An 0.25 mL aliquot sample was removed from the liquid nitrogen storage and thawed at 37°C. The content was transferred to two 0.5 mL microcentrifuge tubes, one would be used for acquiring spectra before antimicrobial incubation and the other one would be for after antimicrobial incubation. Cell pellet was collected by centrifuging at 10,000g for 5 minutes. The skim milk supernatant was removed and 80–100 μL 5% dextrose/saline (freshly prepared and double-filtered by 0.22 μm filter) were used to wash the cell pellet 2–3 times.

To obtain spectra before antimicrobial incubation, *immediately* after the last wash the cell pellet was dispersed in 4 μL 5% dextrose/saline. All liquid contents were transferred to the Si ATR crystal surface using 1 μL pipette tips in 7–8 evenly distributed droplets along the length of the crystal. The added 4 μL liquid helped to lift the cell pellet off the microcentrifuge tube wall. The total sample volume (4 μL + cell pellet) should be 8–10 μL. An identical Si ATR element was placed over the sample to form a “sandwich”. The sample was smeared into a thin film of liquid with the top crystal. The smeared sample would dry up in air in 1–2 minutes, forming a thin, uniform layer of dried bacterial cells. The ATR element was inserted into the HATR accessory and a set of five ATR spectra was acquired.

An example of the dried bacterial film on ATR crystal is shown at the bottom of S2 Fig. ATR spectra acquired from this thin, dried bacterial film on the crystal, as well as the observed antimicrobial-induced bacterial spectral changes, were robust and reproducible, as exampled by the ATR spectra of *E*. *cloacae* BAA2341 and *S*. *aureus* 29213 (MSSA) before and after incubation with antimicrobial (levofloxacin 8 μg/mL and oxacillin sodium 20 μg/mL respectively).

To obtain spectra after antimicrobial incubation, antimicrobial stock solution at 2x concentration was added to the bacterial sample by mixing equal volume of the stock solution with the bacteria sample. The antimicrobial stock solution was prepared within 30 minutes before experiment in 5% dextrose saline and stored at 2–8°C until use. Then the mixture was incubated at 35°C for 4 hours without agitation. After incubation, the cell pellet was collected, washed. ATR spectra were then acquired following the procedure described before.

### ATR spectra preprocessing, building SVMDA models and classification in unknown samples

Each ATR spectrum was subjected to spectral preprocessing before compiling into a spectral reference library. Spectral preprocessing can help to improve classification model sensitivity and specificity by enhancing inherent differences between classes while reducing variation within the sample class. The library was used to train classification models.

Each ATR spectrum was baselined by an “Automatic Baseline Correct” algorithm in the OMNIC software so that spectra from different strains and from before vs. after antimicrobial incubation could be compared with minimal interference from varying baselines. One spectrum was baselined at a time, instead of applying the algorithm over large number of spectra to minimize error introduced due to the automatic choice of the points to baseline. After baseline correction every spectrum was inspected to ensure no artificial peaks were introduced and relative ratio of peaks were preserved, before allowing the spectrum to be added to a reference library that would be used to train and test the machine-learning based classification models. An example showing the raw data (i.e. un-baselined spectra) and the baselined spectra is shown in S4 Fig.

To prepare for training classification model to distinguish the six different bacterial species, the spectra were min-max normalized to ensure the model would “focus” on finding species-specific characteristics instead of the absolute peak intensity differences within the dataset.

We utilized the PLS_Toolbox® from Eigenvector Research, Inc (Mason, MA), a chemometric toolbox on MATLAB platform, to build our classification model. We first built and optimized a partial least square discriminant analysis (PLSDA) pre-model. PLSDA is a “supervised” version of principal component analysis, i.e. class ID are requested from the user and utilized to decide class boundary. We examined the pre-model to ensure genuine differences existed between classes. Then a sum vector machine discriminant analysis (SVMDA) classification model was built based on the PLSDA results. SVMDA is a model optimization technique; the class boundary SVMDA is non-linear to allow better classification for data points that are too close to the boundary in the PLSDA pre-model. The curviness of boundary is governed by the data points closest to the boundary (i.e. “support vector”). The SVMDA model parameters were fine-tuned and compiled, and the analytical sensitivity and specificity were calculated. A more detailed description of our rational of selecting machine learning algorithms as well as the pros and cons of each algorithms is provided in the Supplementary Information section.

To test the performance of the SVMDA model, ATR Spectra from 4 different unknown samples were acquired and baseline/normalized by methods described above. The model performance was assessed by determining the % of data from these unknow sample that were correctly classified the model.

## Results and discussion

### Specificity: Utilize antimicrobial-induced bacterial cell spectral changes to distinguish MDR and susceptible strains

We obtained robust and reproducible ATR spectra (S1 Fig) of dried cell pellet at 10^9^–10^12^ cfu/mL before and after antimicrobials incubation for each bacteria strain, as shown in Fig 1. Multiple measurements on at least two different dates were performed for each bacterial strain, and we compared the results to ensure that the antimicrobial-induced bacterial spectra changes were robust and reproducible. An example demonstrating this robustness is shown in S2 Fig.

10^9^–10^12^ cfu/mL was used because the size of the cell pellet at this concentration range could be readily obtained by centrifugation and handled by pelleting in our laboratory. These spiked bacterial samples were prepared in 5% dextrose/saline, to allow us to obtain high-resolution ATR spectra and study the bacterial spectral feature with minimal interference from the solvent and other contaminants in the sample.

We then compared our results with literature to understand the assignment of functional groups associated with major peaks in the bacterial spectrum. Our ATR bacterial spectra from 4000–1500 cm^-1^ are consistent compared to literature [52–57]. Most of these peaks are vibrational bands of protein and fatty acid, likely part of the cell wall components due to their proximity to the ATR crystal surface. This means that achieving strain specificity with ATR technology is possible since each strain will likely have unique membrane protein and fatty acid compositions. It also means that changes to bacterial cell membrane, such as those induced by interaction with antimicrobial, would be detectable by ATR.

**Fig 2:**
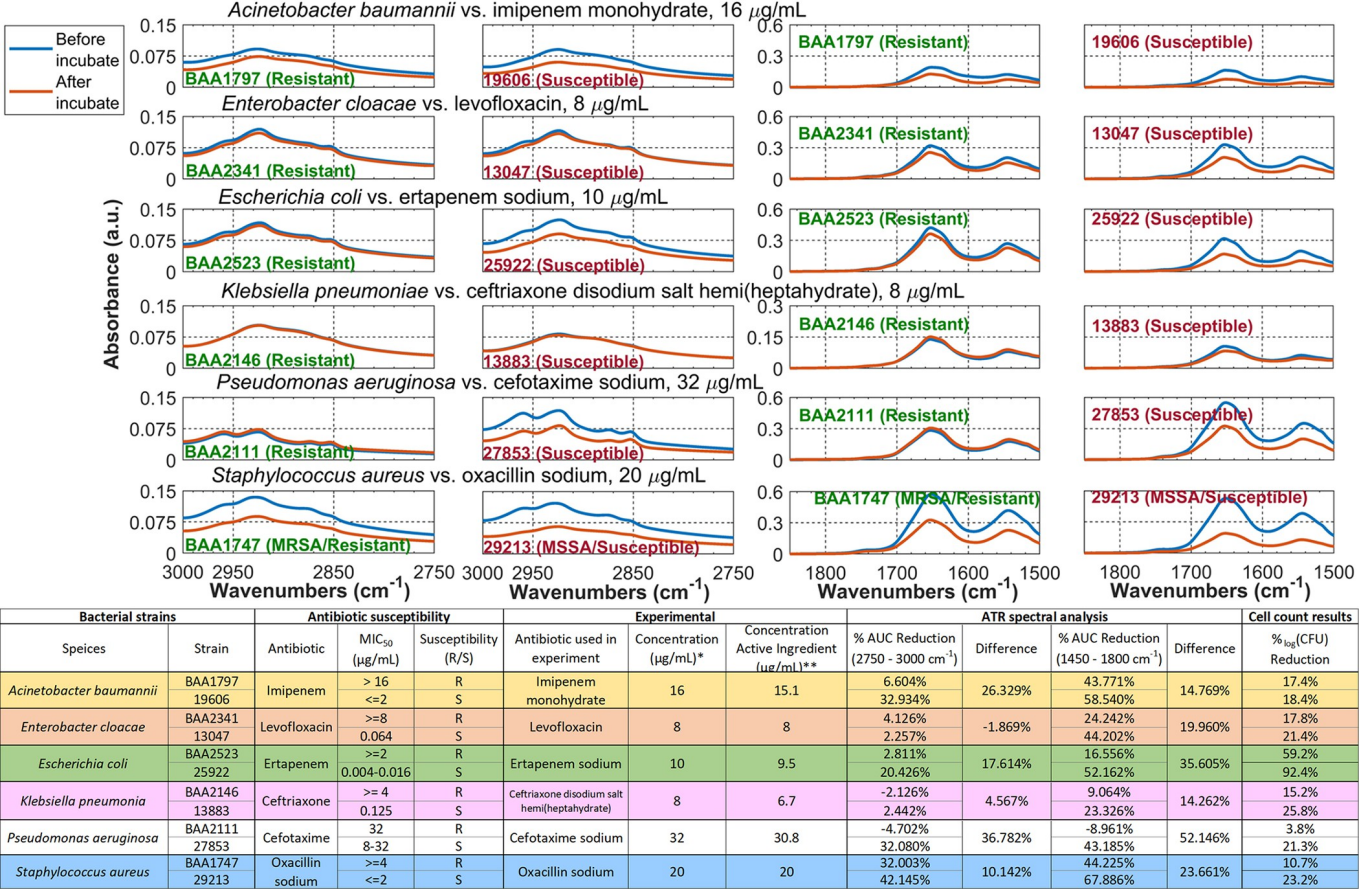
(Top) Zoom-in view to the two highlighted regions in each strain from Fig 1 to show the common spectral change trend between MDR and antimicrobial-susceptible strains in six species. (Bottom) Table summary (1) Strain designation, antimicrobial susceptibility to six different antimicrobials, MIC_50_ values and the sources for the MIC_50_ values. (2) Reagent used in experiment and the concentration of active ingredient. * All antimicrobial solution is prepared in 5% dextrose saline. **Estimated concentration of antimicrobial in the prepared solution. (3) Area Under the Curve (AUC) calculation for each spectrum. (4) # of viable colonies determined by quantitative overnight culture for each strain, before and after antimicrobial incubation.

In Fig 1 we observed peak shifts and peak intensity changes common to all 12 strains, in two spectral regions as highlighted by the red and blue rectangular boxes. Fig 2 shows a zoom-in view of these two regions. The intensities of these two bacterial ATR spectral features are estimated by calculating their area under the curve (AUC). AUC is estimated for the region bounded by the spectrum and the black dotted line, as exampled in S4 Fig. The beginning and the end of the black dotted line were identical for every spectrum in all 12 strains (3000 and 2750 cm^-1^, 1750 and 1500 cm^-1^) to ensure that AUC results from all 12 strains can be compared, in order to find any common trends exist among the MDR strains that can be used to distinguished from the susceptible ones, and correlate to the number of viable cells in sample.

The following definitions were used during the quantitative comparison.

- % AUC reduction = (AUC_MDR_−AUC_susceptible_)/(AUC_MDR_) x 100%.
- CFU = colony forming unit. %_log_CFU reduction = (log CFUbefore−log CFU_after_)/(log CFU_before_) x 100%.

The quantitative spectral analysis results are shown in the table at the bottom of Fig 2. The analysis reveals the following two general trends (1) susceptible strain have larger % AUC reduction than the MDR one in all six bacterial species in both spectral regions, and (2) %AUC reduction can be correlated to the number of viable cells as measured by %_log_CFU reduction. Larger % AUC reduction corresponds to larger %_log_CFU reduction and vice versa. For example, the levofloxacin-resistant *E*. *cloacae* BAA2341 has 24.242% AUC reduction for the 1800–1450 cm^-1^ region, while the susceptible *E*. *cloacae* 13047 has 44.202% reduction in the same region. Correspondingly, the number of viable cells decreased after antimicrobial incubation is greater in *E*. *cloacae* 13047 than in *E*. *cloacae* BAA2341 (21.4% vs. 17.8%, respectively).

We had verified that these peak intensity changes were entirely due to change on the bacteria cell instead of spectroscopic contributions from the added antimicrobial solutions as shown in S3 Fig.

Figs 1 and 2 shows that our ATR method can detect changes due to antimicrobial-induced bacterial cell changes spectroscopically and uses these changes to distinguish MDR and susceptible strains.

### Selectivity: Detect bacterial spectral features in mixtures

Combat-related wounds are often contaminated by foreign materials at the point of injury (for example, dust and soil, cloth fiber from bandage and bed linen, metal pieces, and so on) [7,58]. In some infections a possibility of multiple strains can be presented [4,9]. Infected wound samples are typically collected in the form of swab, pus aspirates and tissue/blood biopsy samples [59], and the choice of sampling technique depends on the condition of the patient, resources available and the type of wounds. Therefore, samples from infected wounds are highly complex mixtures, and one of the most important capability of the diagnostic system is the ability to recognize each bacterial species spectral features in the mixture.

The potential of our ATR technology in detecting bacterial features in mixtures is demonstrated below by acquiring high-quality ATR spectra from two bacteria-spiked, protein-rich mixtures.

#### Example 1: recognize bacterial spectral features in protein-rich mixture

**Fig 3:**
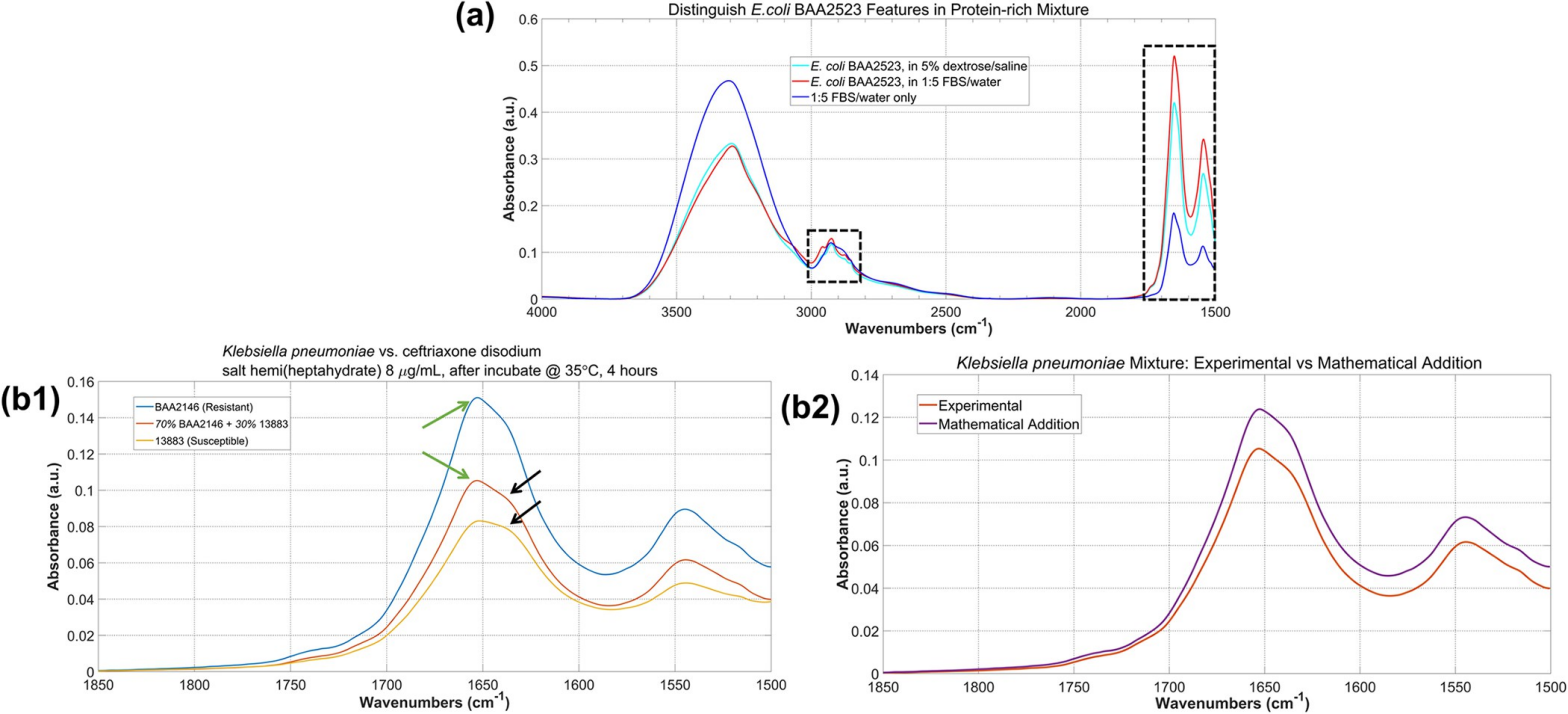
Selectivity of the ATR system. (a) averaged ATR spectra of *E*. *coli* BAA2523 in (cyan) 5% dextrose saline and in (red) FBS/water 1:5 mixture. The spectra of the FBS/water 1:5 mixture itself is shown in Blue. The two black rectangular boxes highlight two spectral regions where clear spectral differences can be seen. (b1) ATR spectra of *K*. *pneumoniae* BAA2146 (MDR), *K*. *pneumoniae* 13883 (susceptible) and a mixture of 60% BAA2146 and 40% 13883. Zoom-in view to the Amide I–Amide II spectral region (1500–1850 cm^-1^). # of viable cells in all three are in the 10^7^ cfu/mL range. (b2) Contributions from BAA2146 and 13883 are clear in the mixture spectrum, marked by similarity between the experimental spectrum and the mathematical addition of the BAA2146 and 13883.

Fig 3(A) shows an average of five spectra of *E*. *coli* BAA2523 in a fetal bovine serum/water (FBS, Sigma-Aldrich®) 1:5 (v/v) mixture. Compared to the *E*. *coli* BAA2523 spectrum in 5% dextrose/saline from Fig 1, the bacterial features were clearly distinguishable in the protein-rich mixture, as shown in the two regions highlighted by the black boxes.

#### Example 2: distinguish MDR and susceptible strain in the same mixture

We obtained ATR spectra of a mixture sample consisted of 60% *Klebsiella pneumoniae* BAA2146 (MDR) and 40% *Klebsiella pneumoniae* 13883 (susceptible), and successfully recognized both strains in the mixture. The spectra of the *K*. *pneumoniae* BAA2146 sample, the *K*. *pneumoniae* 13883 sample and the 60/40 mixture showed very similar spectral features after incubation with 8 μg/mL ceftriaxone disodium solution for 4 hours at 35°C. When zoom-in to the 1500–1850 cm^-1^ region (where Amide I and Amide II vibrational features are), all three samples show different relative ratio of the ~1660 and ~1630 cm^-1^ peaks marked by the green and the black arrows, respectively, as shown in Fig 3(B1). The mixture spectrum shows clear contribution from both strains. Quantitative analysis on the mixture spectrum reveals more contribution from *K*. *pneumoniae* BAA2146 and less from *K*. *pneumoniae* 13883, characterized by the similarity between the experimental mixture spectrum and the mathematical addition of 60% *K*. *pneumoniae* BAA2146 spectrum + 40% *K*. *pneumoniae* 13883 spectrum, as shown in Fig 3(B2).

In summary, Fig 3 shows the selectivity of our ATR system to recognize high-resolution, strain-specific bacterial spectral feature in complex mixture, and use these features to distinguish MDR and antimicrobial-susceptible strains.

### Sensitivity: Observe antimicrobial-induced bacterial cell spectral changes at 10^4^ cfu/mL

Figs 1–3 demonstrated the capability of our ATR system to recognize antimicrobial-induced spectral changes and discern MDR and antimicrobial-susceptible strains in cell pellet samples (10^9^−10^12^ cfu/mL). In this section, we demonstrate similar capability of our ATR system at much lower cell concentration of 10^4^ cfu/mL.

Experimentally, two identical 50 mL samples of ertapenem-susceptible *E*. *coli* 25922 at ~10^4^ cfu/mL in 0.85 wt% NaCl solution were prepared. The bacteria cells were collected by filtering the solution through a 0.22 μm PTFE membrane filter using a 47 mm glass vacuum filtration assembly. The membrane filter was air-dried and ATR spectra of the cells on the membrane were acquired by pressing the membrane filter against the ATR element with a pressure-clamp system. We verified that no viable bacteria cells had pass through the membrane filter in independent experiment, by observing no bacteria colony growth in the liquid after filtration.

Ertapenem sodium was added to one of the two samples to make the final concentration of 10 μg/mL, and the sample was set for 4 hours at 35°C with agitation. Spectra were acquired after the filtration of the sample. For the other sample, filtration and spectral acquisition were done immediately after preparation.

**Fig 4:**
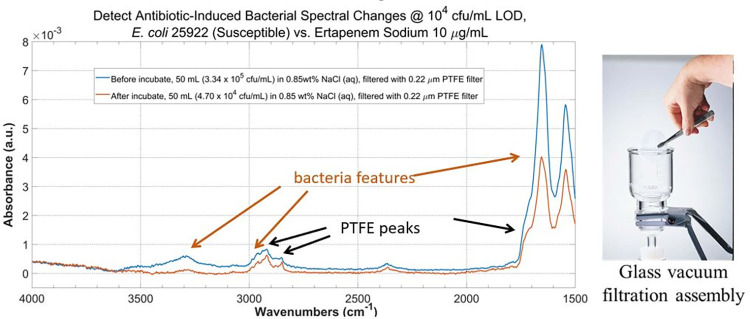
Sensitivity of the ATR system. Antimicrobial-induced bacterial spectral changes can be detected at 10^4^ cfu/mL for an ertapenem-susceptible *E*. *coli* 25922 samples before and after incubation with 10 μg/mL ertapenem sodium at 10^4^ for 4 hours at 35°C.

The ATR spectra before and after antimicrobial incubation with this filtration + ATR procedure is compared in Fig 4 (left). Due to the size of the filtration assembly (Fig 4 right, 47 mm diameter), 50 mL sample volume was needed. With 10^4^ cfu/mL bacteria cell concentration, we expected that only a small fraction of the PTFE membrane will be covered by the bacterial cells. Therefore, some of the PTFE spectral features (marked by the black arrows) are observed along with the bacterial features (marked by the green arrows). However, bacterial features as well as the antimicrobial-induced peak intensities reduction are clearly distinguishable from the PTFE features.

From Fig 4 we noticed that the overall bacterial peak intensities were weak (in the order of 10^−3^ range). To investigate possible strategies to improve the bacterial signal intensity, as well as further verify the ability of this filtration + ATR procedure to observe the antimicrobial-induced bacterial spectral changes at low concentration, we repeated the experiment on the levofloxacin-resistant *E*. *cloacae* BAA2341 using a 0.22 μm polycarbonate (PCTE) membrane filter on the 47 mm glass vacuum filtration assembly we used in Fig 4.

**Fig 5:**
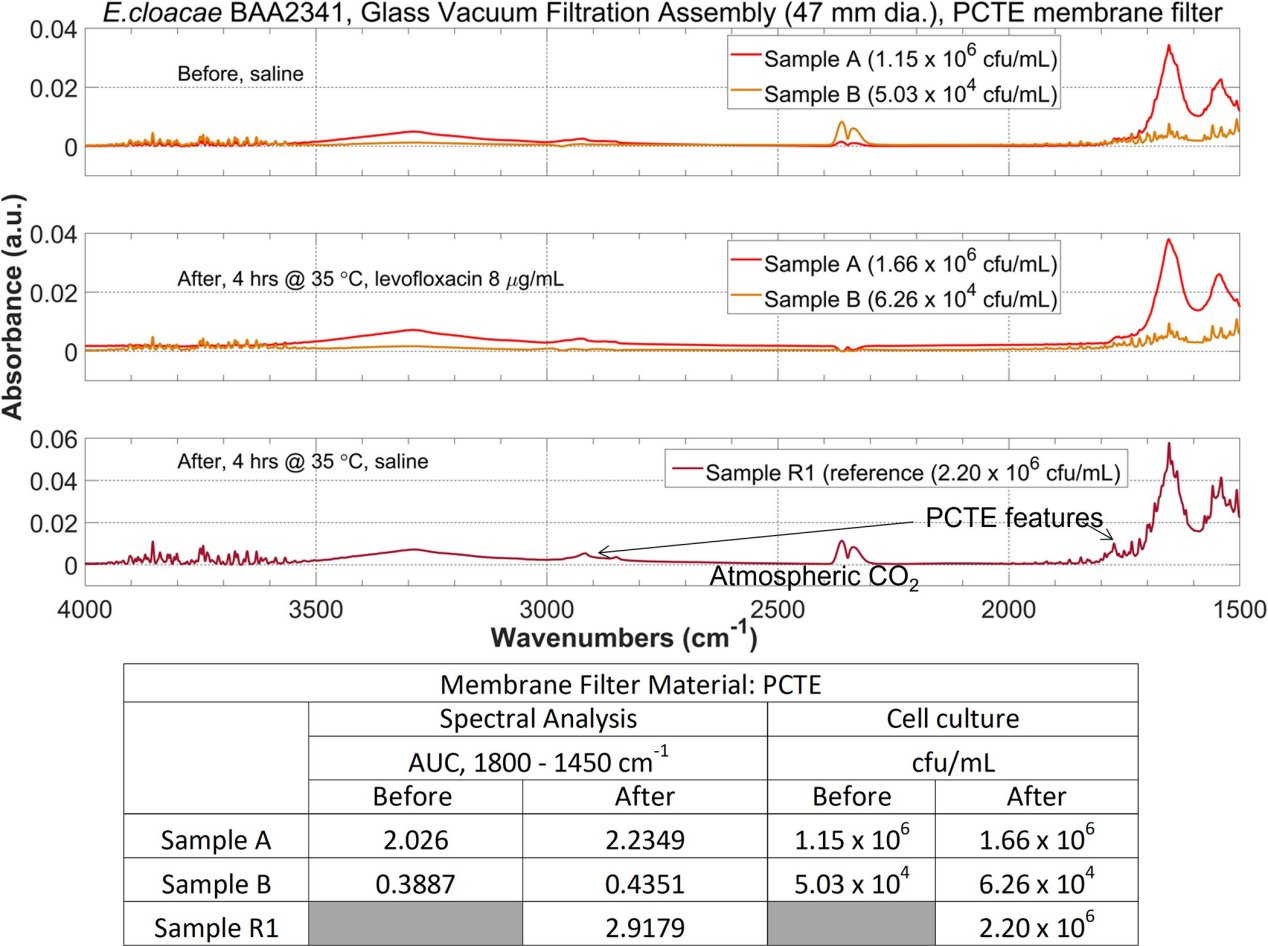
Antimicrobial-induced bacterial spectral changes at 10^6^ and 10^4^ cfu/mL can be detected for a levofloxacin-resistant *E*. *cloacae* BAA2341 samples before and after incubation with 8 μg/mL levofloxacin for 4 hours at 35°C. A sample of bacteria incubated in saline under the same temperature for the same duration was prepared as positive control and ATR spectra were acquired. The bacteria peak intensities in the before and after incubation spectra as well as from the positive control spectrum did not vary significantly, which were confirmed by the cell culture results and matches with the levofloxacin-resistance nature of this bacteria strain.

The experimental procedure was identical as described above, except the sample volume was 30 mL instead of 50 mL. We prepared two samples at ~10^6^ and 10^4^ cfu/mL concentration level. A positive control (a sample in 0.85% NaCl that were incubated for the same amount of time under the same temperature as the one with antimicrobial) was prepared and its cell concentration was determined by cell culture. The ATR spectra for this *E*. *cloacae* BAA2341 are shown at the top in Fig 5 and the AUC calculation is shown at the bottom. Since this bacteria strain is resistant to the antimicrobial (levofloxacin 8 μg/mL), the cell concentration is not expected to decrease significantly after incubation with the antimicrobial. This is reflected by the similar AUC values at the bacterial peak at1800–1450 cm^-1^ region on the before incubation and after incubation spectra. Similarly, bacteria peak on the positive control spectrum has similar intensity as the one on the before incubation spectrum, because bacteria concentration did not change significantly.

Hence, Figs 4 and 5 demonstrated the ability to observe antimicrobial-induced bacterial spectral changes at 10^4^ cfu/mL from both antimicrobial-susceptible *E*. *coli* 25922 and antimicrobial-resistant *E*. *cloacae* BAA2341 sample with our ATR + filtration strategy.

Fig 5 also showed a path for future improvement of bacteria signal intensity that may help leading to lower detection limit. Compared to the spectra in Fig 4, the bacteria signal in Fig 5 is almost one order of magnitude higher at similar bacteria concentration level. We hypothesized the reason for this improvement is related to the nature of the PCTE membrane. PCTE membrane filter is manufactured using a process called track-etching (“PCTE” = polycarbonate track-etched), producing very small pore size distribution, i.e. all pores on the filter have virtually the same size [60]. On the contrary, PTFE membrane is produced via sintering process and therefore have greater pore size variations. As a result, most bacteria cells will remain on the *surface* of the membrane filter and be interrogated by the evanescent field on the ATR crystal surface instead going into the matrix of the membrane filter, when filtering by PCTE membrane filter with pore size smaller than the averaged cell size.

### Automation: Machine learning for objective, quantitative spectral analysis and classification

**Fig 6:**
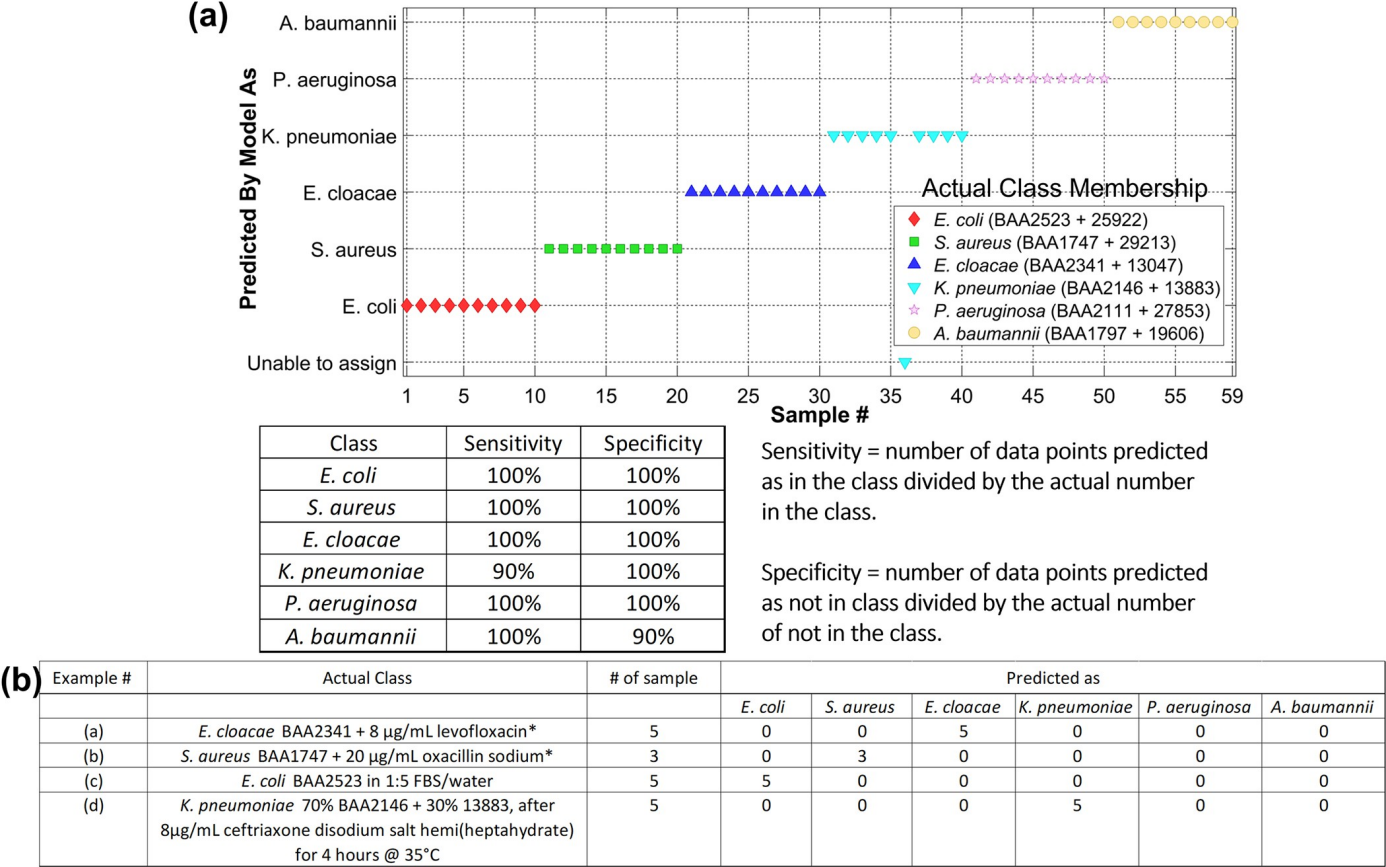
Automatic spectral analysis by machine learning models (a) >95% species specificity and sensitivity achieved in distinguishing six bacterial species based on ATR spectral features. (b) numerical summary of classification results by the machine learning model for four different unknown samples.

We built a sum vector machine–discriminant analysis (SVMDA) model using the ATR spectra reported in Fig 1. The five ATR spectra from the MDR and the susceptible strains after antimicrobial incubation in each species are combined to form one class. The results are shown in Fig 6(A). Each dot in the figure represents one spectrum. The class membership ID predicted by the model is shown in the y-axis, and the actual class membership (i.e. the “correct” answer) is shown in the legend. Each species is color-coded.

Our SVMDA model achieved >95% averaged analytical sensitivity and specificity for the six bacterial species. To test the performance of our SVMDA model, we applied ATR spectra from four different samples, including the ones shown in Fig 3, and recorded the species identity classified by the model. The results are summarized numerically in Fig 6(B). Every spectrum in these four unknown samples were correctly classified by the model.

## Conclusion

In conclusion, we demonstrated the potential of our RAPID system based on FTIR-ATR to detect and distinguish MDR strains in pure solution and in mixture, by recognizing unique bacterial ATR spectral features and antimicrobial-induced changes of these features. We demonstrated a 10^4^ cfu/mL detection limit and a machine learning model with >95% averaged analytical sensitivity and specificity, as well as 100% accuracy in classifying species identity in four unknown samples. Our results clearly show our RAPID system ATR technology can meet the critical need for a rapid portable system suitable for front line users to detect the presence of MDR bacteria in infected combat-related wounds.

## Supporting information

S1 Fig Robust (i.e. small variation between samples in the same dataset) and reproducible (i.e. small variation between datasets acquired during different trials) bacteria ATR spectra for *E*. *cloacae* BAA2341, *E*. *cloacae* 13047, *E*. *coli* BAA2523 and *S*. *aureus* 29213 from multiple trials. Showing five individual spectra that were used to calculate the averaged spectrum in Fig 1. Showing the ATR spectra from two different trials.
(TIF)

S2 Fig (Top and Middle) Robustness and reproducibility of the observed antibiotic-induced ATR bacterial spectral changes. Exampled by the five individual *E*. *cloacae* BAA2341 and *S*. *aureus* 29213 before and after antibiotic incubation spectra, which were used to calculate the averaged spectrum in Fig 1. Showing the ATR spectra from two different trials. (Bottom) the robustness and reproducibility were achievable by smearing the bacteria pellet onto the ATR crystal surface, resulting in a thin, uniform film on the surface after air-drying, as exampled in this figure.
(TIF)

S3 Fig Compare averaged ATR spectra of the bacteria cell before and after incubation with antimicrobial solution at 35°C for 4 hours. The spectrum from the antimicrobial solution used in the experiment are shown in black for each strain.
(TIF)

S4 Fig Averaged ATR spectrum before and after application of the Automatic Baseline Correct algorithm (within the OMNIC® software package from Thermo Fisher®). The baselined spectrum is used to estimate the bacterial peak intensity (the broad peaks between 3000–2750 cm-1 and 1750–1500 cm-1) by calculating the area under the curve (AUC). AUC is estimated for the region bounded by the spectrum and the black dotted line.
(TIF)

S5 Fig (Left) PCA model can distinguish the *E*.*coli* and *S*. *aureus* classes. Each dot represent a spectrum. The colored circle is the 95% confidence interval of the class. (Right) as more classes is added (the *E*. *cloacae* class), it overlapped with the existing classes.
(TIF)

S6 Fig (Top) Min-Max normalized spectra for *Enterobacter cloacae* BAA2341 and 13047. (Middle) 2^nd^ derivatives of the Min-Max normalized spectra. (Bottom) Zoom-in view to show that subtle spectral differences become more obvious in the two highlighted spectral regions.
(TIF)

S7 Fig The use of nonlinear decision plane allows more data points to be corrected classified in a hypothetical two-class model. (TIF)
