# Supplementary figures and images for: Rapid, label-free pathogen identification system for multidrug-resistant bacterial wound infection detection on military members in the battlefield
Source: PLoS One. 2022 May 5;17(5):e0267945. doi: 10.1371/journal.pone.0267945 (PMC9070933; doi:10.1371/journal.pone.0267945)

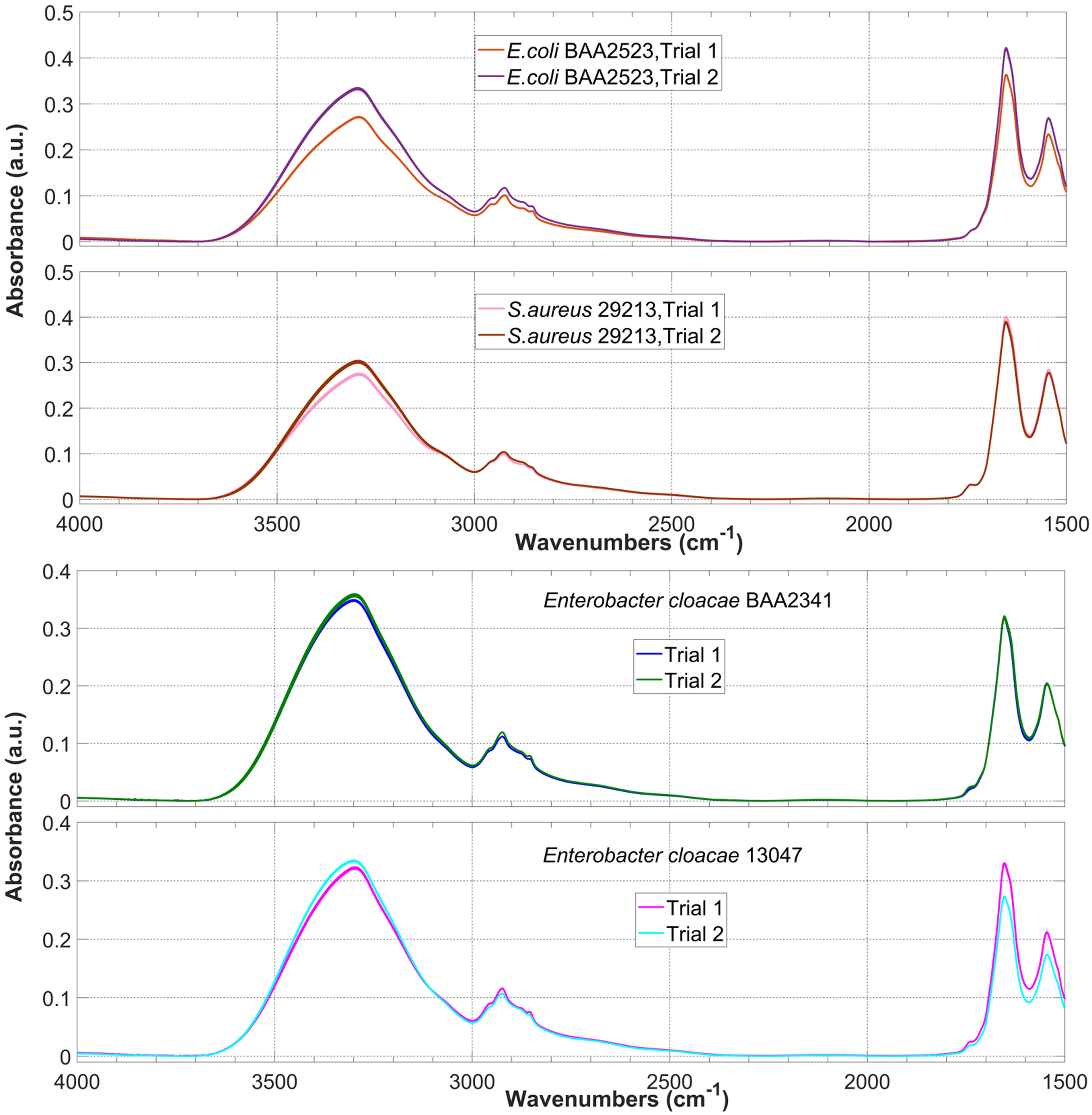

Supplement: S1 Fig — Showing five individual spectra that were used to calculate the averaged spectrum in Fig 1. Showing the ATR spectra from two different trials. (TIF) [file pone.0267945.s001.tif]

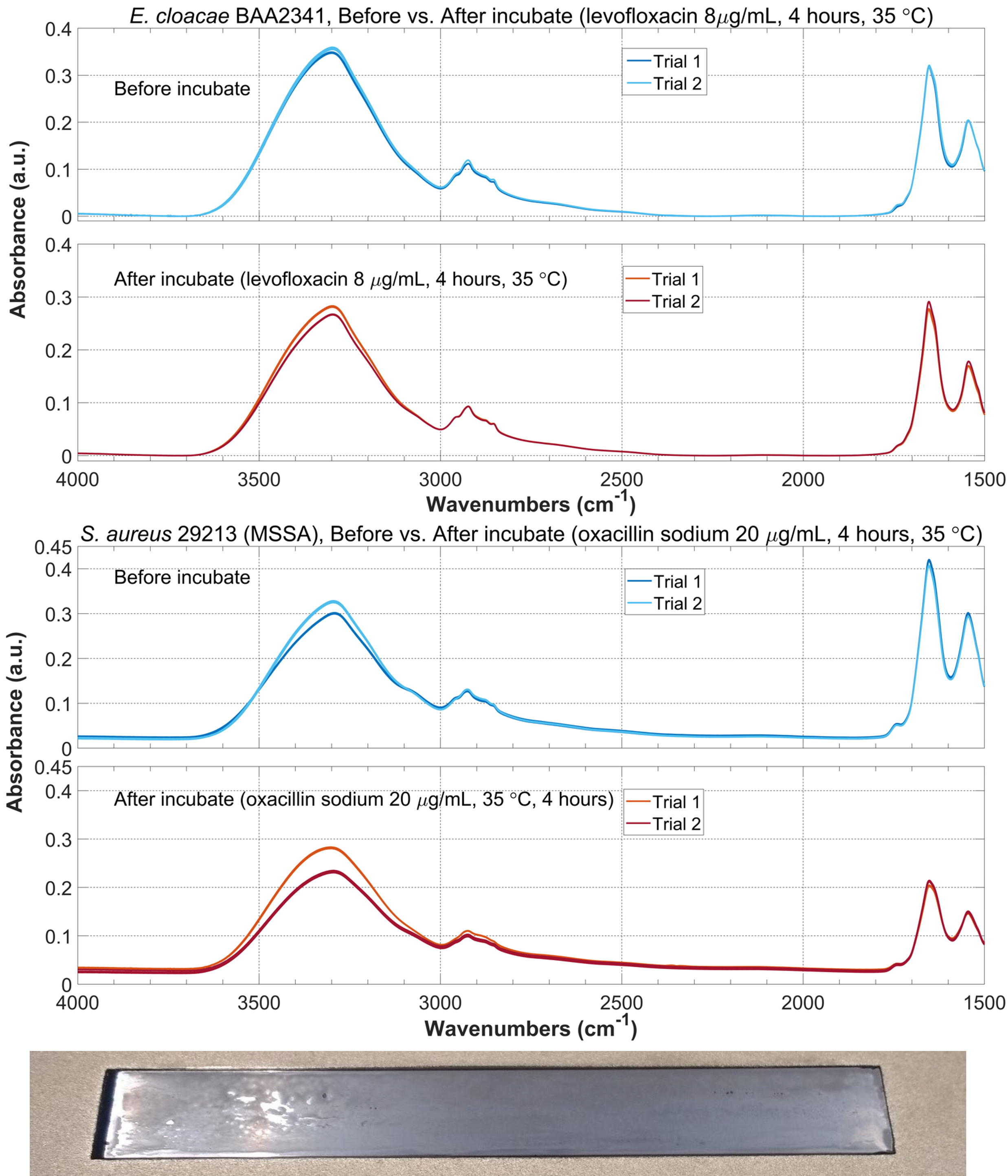

Supplement: S2 Fig — (Top and Middle) Robustness and reproducibility of the observed antibiotic-induced ATR bacterial spectral changes. Exampled by the five individual E. cloacae BAA2341 and S. aureus 29213 before and after antibiotic incubation spectra, which were used to calculate the averaged spectrum in Fig 1. Showing the ATR spectra from two different trials. (Bottom) the robustness and reproducibility were achievable by smearing the bacteria pellet onto the ATR crystal surface, resulting in a thin, uniform film on the surface after air-drying, as exampled in this figure. (TIF) [file pone.0267945.s002.tif]

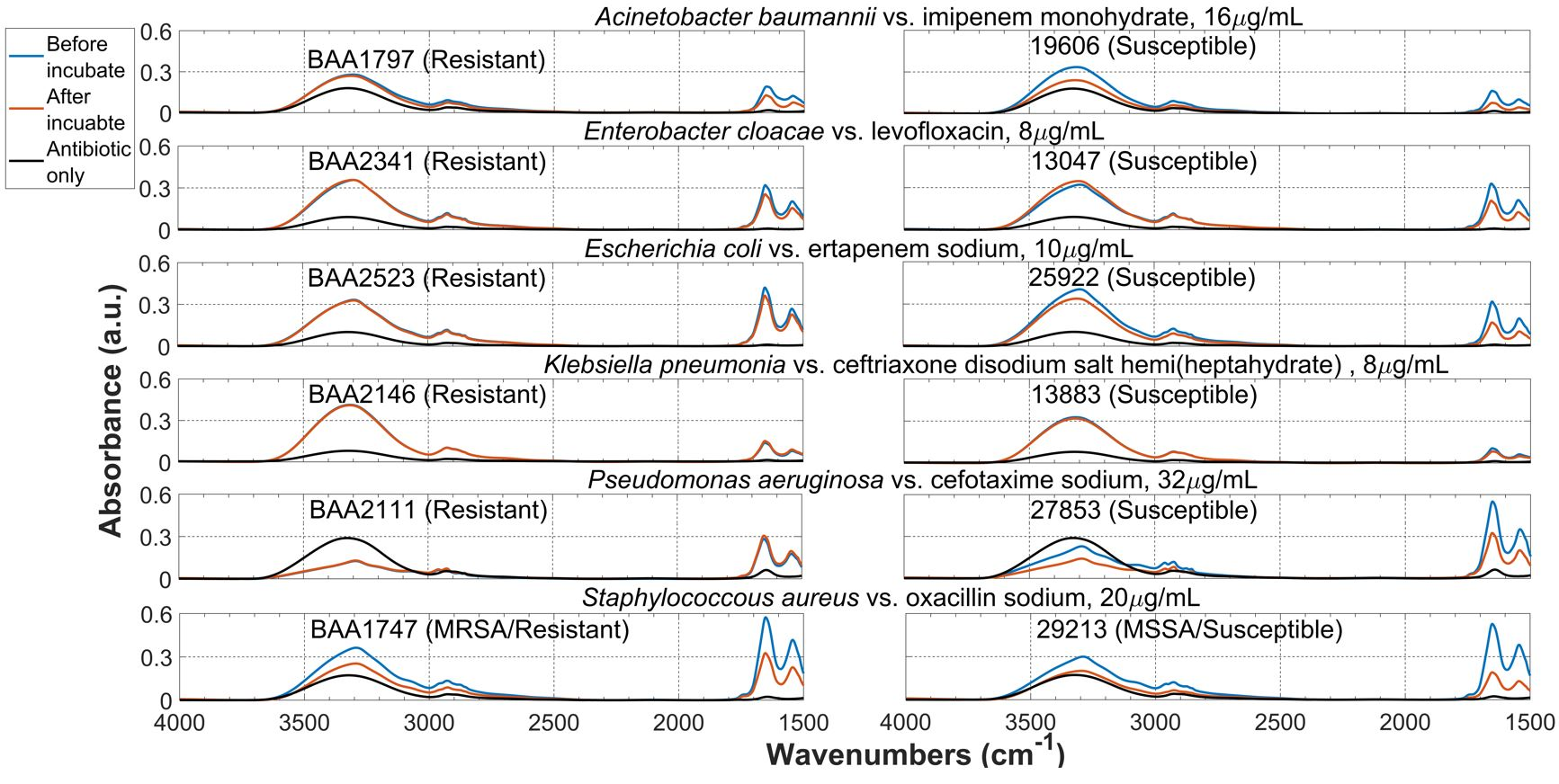

Supplement: S3 Fig — The spectrum from the antimicrobial solution used in the experiment are shown in black for each strain. (TIF) [file pone.0267945.s003.tif]

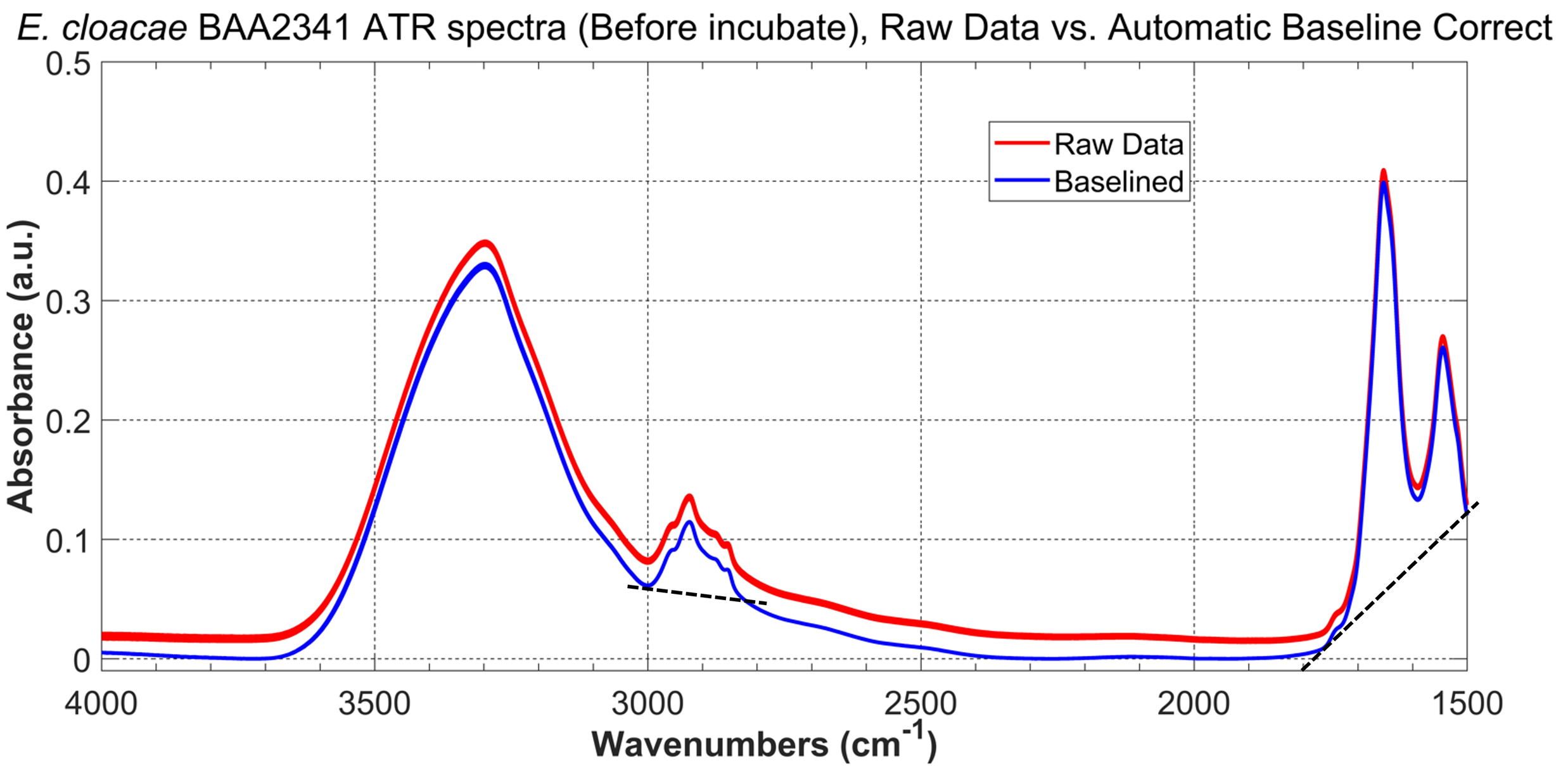

Supplement: S4 Fig — The baselined spectrum is used to estimate the bacterial peak intensity (the broad peaks between 3000–2750 cm-1 and 1750–1500 cm-1) by calculating the area under the curve (AUC). AUC is estimated for the region bounded by the spectrum and the black dotted line. (TIF) [file pone.0267945.s004.tif]

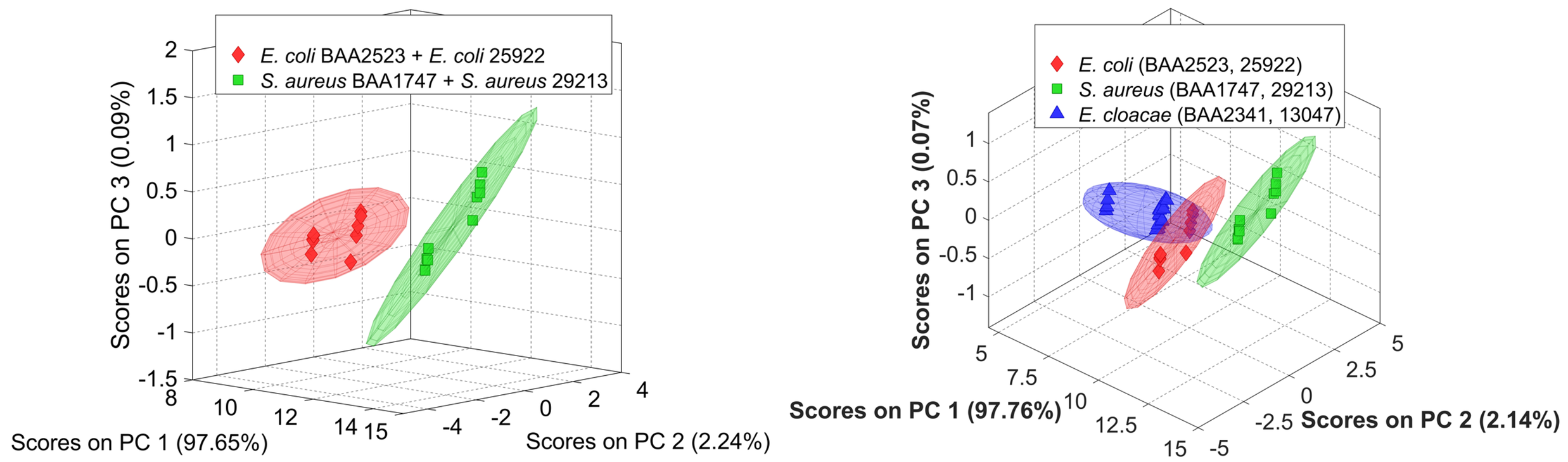

Supplement: S5 Fig — (Left) PCA model can distinguish the E.coli and S. aureus classes. Each dot represent a spectrum. The colored circle is the 95% confidence interval of the class. (Right) as more classes is added (the E. cloacae class), it overlapped with the existing classes. (TIF) [file pone.0267945.s005.tif]

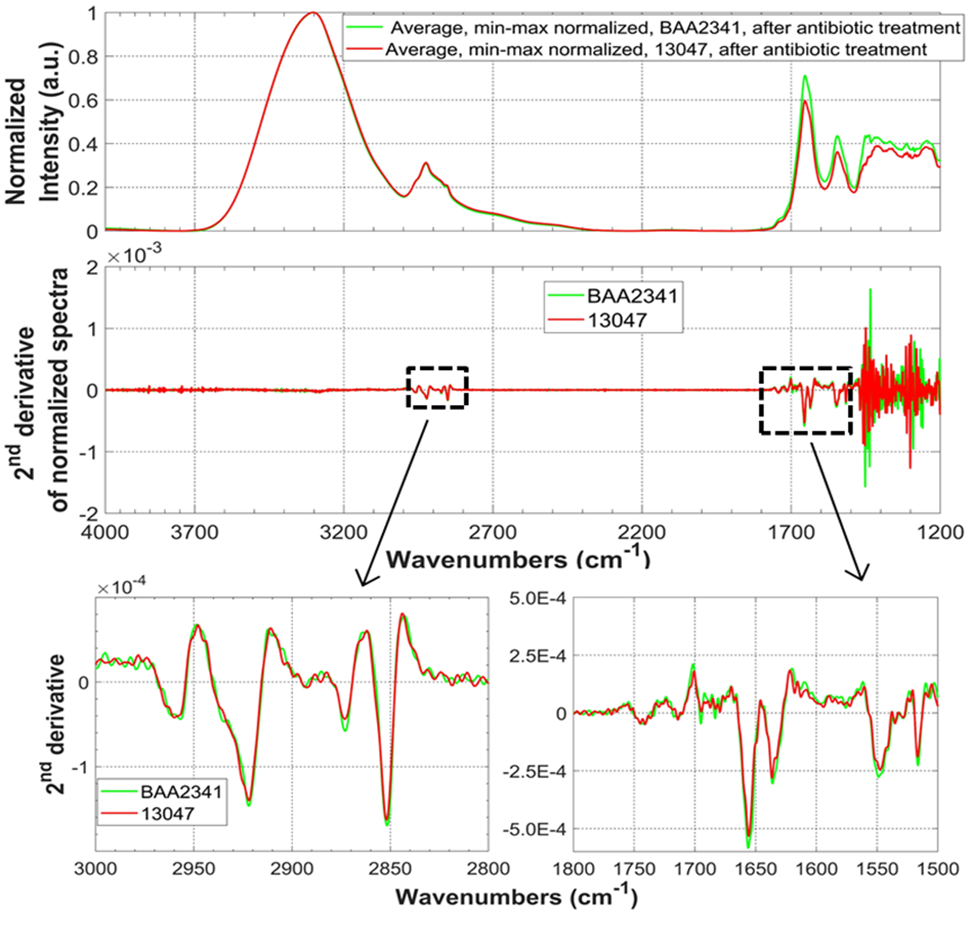

Supplement: S6 Fig — (Top) Min-Max normalized spectra for Enterobacter cloacae BAA2341 and 13047. (Middle) 2nd derivatives of the Min-Max normalized spectra. (Bottom) Zoom-in view to show that subtle spectral differences become more obvious in the two highlighted spectral regions. (TIF) [file pone.0267945.s006.tif]

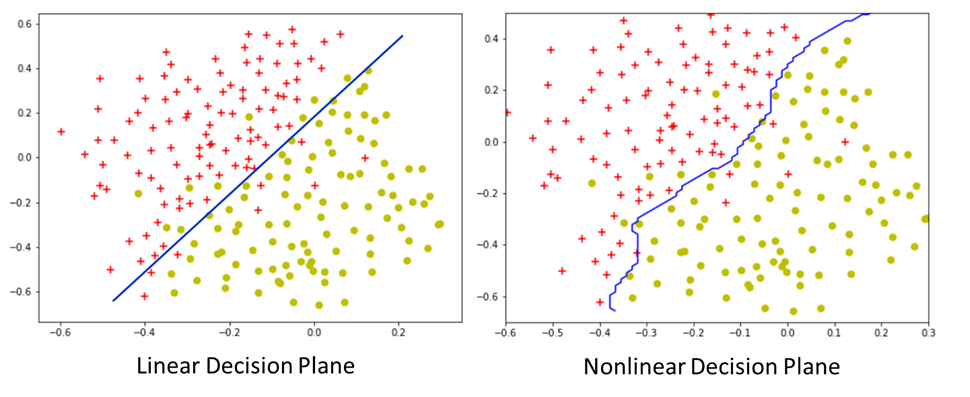

Supplement: S7 Fig — (TIF) [file pone.0267945.s007.tif]
